# Clinical Assessment of Plaque Removal Using a Novel Dentifrice Containing Cellulose Microfibrils

**DOI:** 10.3390/dj12010007

**Published:** 2023-12-29

**Authors:** Mohamed E. Labib, Antonio Perazzo, James Manganaro, Yacoob Tabani, Kimberly R. Milleman, Jeffery L. Milleman, Laurence J. Walsh

**Affiliations:** 1 NovaFlux, Inc., Princeton, NJ 08540, USA; perazzo@novaflux.com (A.P.); manganaro@novaflux.com (J.M.); tabani@novaflux.com (Y.T.); 2 Salus Research Inc., Fort Wayne, IN 46825, USA; kmilleman@salusresearch.us (K.R.M.); milleman@salusresearch.us (J.L.M.); 3 School of Dentistry, The University of Queensland, Herston 4006, Australia; l.walsh@uq.edu.au

**Keywords:** oral hygiene, dentifrices, dental plaque, bacterial biofilm, micro-fibrillated cellulose

## Abstract

Dentifrice performance in the removal of dental plaque is influenced by the interactions of dentifrice components with tooth surfaces. This randomized controlled clinical study assessed the effectiveness and safety of a novel fluoride dentifrice formulation that included a micro-fibrillated cellulose network with entangled microcrystalline cellulose and silica particles (Protegera^TM^), and compared this to a positive control fluoride dentifrice (Crest Cavity Protection™). Whole mouth dental plaque levels in 82 healthy adults were measured after the first supervised use, and following a week of twice daily use at home. Overall, the test dentifrice was at least three times and up to four times more effective in whole-mouth plaque reductions, with a 38.6% reduction on first use, a 30.9% reduction at day 7, and a 41.6% reduction from day 1 to day 7, compared to reductions of 12.0%, 9.6% and 11.6%, respectively for the positive control, and up to seven times more effective in lingual plaque reductions, than the reference dentifrice (*p* < 0.001), with a 27.7% reduction on first use, a 22.3% reduction at day 7, and a 31.0% reduction from day 1 to day 7, compared to reductions of 4.4%, 2.2%, and 4.5%, respectively, for the positive control. No safety issues arose from the use of the test dentifrice. These results indicate that including micro-fibrillated cellulose enhances plaque removal effectiveness, without causing adverse changes to oral soft tissues.

## 1. Introduction

Essential self-performed oral health behaviors include twice daily mechanical toothbrushing using a fluoride dentifrice and interdental cleaning [1,2]. While toothbrushing has value for preventing dental plaque-induced oral diseases [3,4,5], many patients struggle with effectively performing mechanical tooth cleaning [6], and this has prompted the development of powered brush technologies, and enhancements to the formulation of fluoride dentifrices. Despite such improvements, it is common for patients to have dental plaque deposits remaining in most regions of the dentition upon the completion of a toothbrushing episode [7,8,9,10,11,12,13].

A contributing factor to the less-than-ideal performance of modern toothbrushes and dentifrices is that the collective mechanical action of a toothbrush with a dentifrice may be less than ideal, in terms of the forces that are generated on the tooth surfaces where plaque deposits are present [14,15]. A specific issue is the interaction of tooth-brush bristles with the tooth surface. Viscosity modifiers (including glycerol and polymeric thickeners) and detergents may act as lubricants between dental plaque deposits and toothbrush bristles, thus limiting the mechanical removal of dental plaque [14,15,16,17]. This lubricating effect is enhanced by stimulated saliva that dilutes the dentifrice within the oral cavity, creating a slurry that does not have suitable mechanical properties to remove plaque.

Given these considerations, it is not surprising that numerous studies have demonstrated that dentifrices are ineffective in improving the action of the toothbrush in mechanically removing plaque biofilm [7,8,9,10,11,12,13]. The 2016 systematic review undertaken by Valkenburg et al. [13] included 20 comparisons of toothbrushing with a dentifrice compared to brushing without a dentifrice. The extent of plaque removal from brushing alone was on average 50.3%, while brushing with a dentifrice achieved a reduction of just 49.2%. Several meta-analyses confirmed that there is no significant difference when toothbrushing with and without a dentifrice.

To enhance the plaque removal capabilities of a dentifrice, one approach is to include a material made of fibrils that when formulated as a dentifrice forms a microstructural network with tailored mechanical properties [18,19] which can create the shear stress needed to remove dental plaque as it moves across the surface of dental plaque deposits. This allows the dentifrice to remove plaque even in dental areas not reached by the brush bristles, and makes it less prone to interference from lubricant effects. Such materials can be of natural origin, e.g., micro-fibrillated cellulose (MFC) derived from wood pulp, and can include (micro)fibrils of different thicknesses and lengths. Within these microscale three-dimensionally entangled networks of fibers, abrasive particles such as silica [18,19], microcrystalline cellulose, and polymeric thickeners can be included as needed. Due to the interconnected entangled microstructure, the clusters of microfibrils do not fully break down into a slurry upon saliva-induced dilution, and maintain sufficient mechanical properties that enhance dental plaque biofilm removal [20,21]. Any abrasive particles that are trapped within the fiber network will be dragged along the surface of the tooth as fluid flow occurs, causing some of these to drag against the surfaces being cleaned. This allows the fiber networks to make direct contact with the plaque biofilm under normal brushing conditions. At the same time, fibers can become entangled with stacks of the biofilm, and cause these to become detached. This technology has been deployed for cleaning channels within medical devices [18,19].

A fluoride dentifrice based on this same concept is Protegera^TM^ (Protegera, Inc., Madison, WI, USA), where the cellulose fibers are derived from the wood pulp of Norway spruce trees using a mechanical process [20,21]. MFC is a “Generally Recognized As Safe” (GRAS) ingredient according to the US Food and Drug Administration and is approved as a food additive. In this dentifrice, MFC fibers form an extended toothpaste-containing matrix that is designed to dislodge deposits of dental plaque, including from regions beyond the reach of toothbrush bristles [20,21]. The water-insoluble MFC fiber matrix protects the incorporated toothpaste ingredients from significant saliva-induced material microstructural breakdown known to occur in traditional toothpaste made with soluble polymers such as carboxymethyl cellulose (CMC), xanthan gum and carrageenan [20,21]. Brushing provides the shear stress needed to force the MFC fiber matrix to dislodge the plaque biofilm from tooth surfaces. MFC has a low relative dentin abrasivity (RDA) score (RDA = 5), and hence the fiber complexes clean tooth surfaces without damaging the enamel, dentin, or oral mucosal tissues [20,21]. MFC does not exert any direct antimicrobial actions.

Since no past studies have explored the clinical performance of a dentifrice containing cellulose microfibrils, the aim of the present study was to assess the plaque removal efficacy and safety of Protegera™ dentifrice immediately after the first, supervised use and following a week of twice, daily use at home, and compare this to a commonly used fluoride dentifrice (Crest Cavity Protection™). The null hypothesis was that there would be no difference between the two dentifrices. 

## 2. Materials and Methods

### 2.1. Study Design

**Figure 1:**
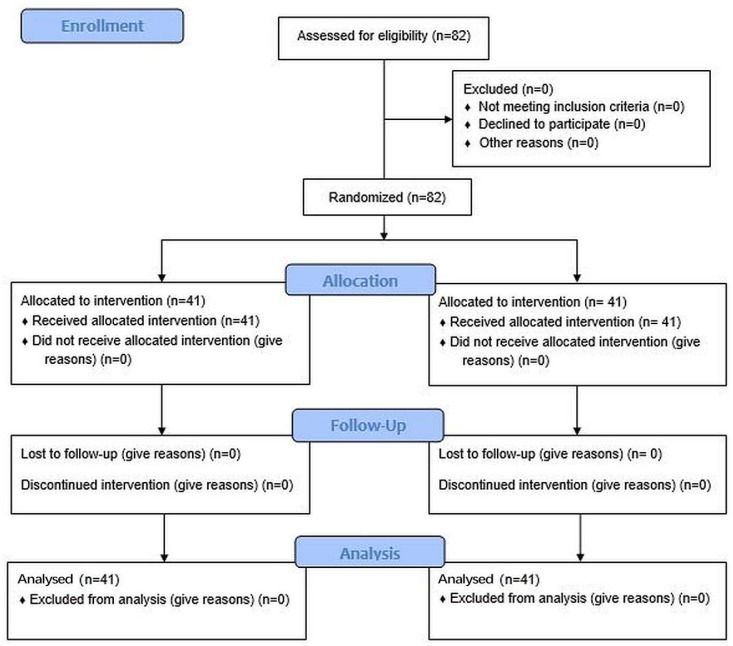
CONSORT diagram.

**Figure 2:**
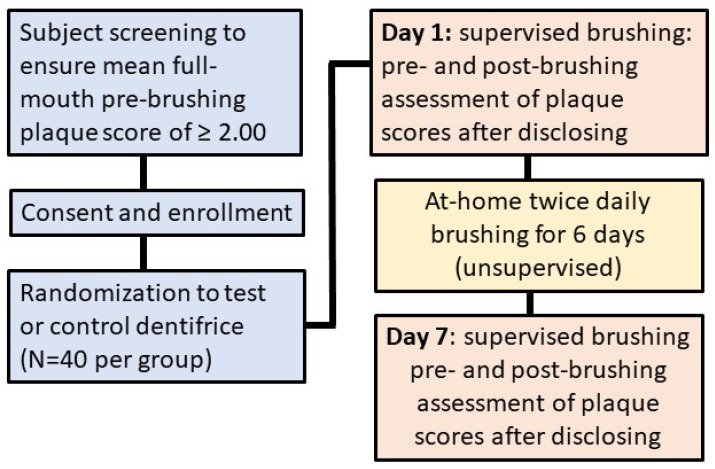
Study design.

The study used a randomized, single-center, examiner-blinded, parallel design. Subjects were randomized 1:1 between the test product (Protegera^TM^ dentifrice, Protegera, Inc., Madison, WI, USA) and the control (Crest Cavity Protection™, Procter & Gamble, Cincinnati, OH, USA) based on a computer-generated randomization schedule prepared in advance of the study. The staff responsible for random assignments were blinded to the treatment protocol. The CONSORT diagram is shown in Figure 1 and the study design in Figure 2.

The control fluoride dentifrice contained 0.243% sodium fluoride (1500 ppm fluoride), with the following additional ingredients: sorbitol, water, hydrated silica, sodium lauryl sulfate, trisodium phosphate, flavor, cellulose gum filler, sodium phosphate, carbomer, sodium saccharin, titanium dioxide, and blue 1 color. The test dentifrice also contained 0.243% sodium fluoride (1500 ppm fluoride), with the following additional ingredients: water, glycerol, hydrated silica, microcrystalline cellulose, micro-fibrillated cellulose, peppermint flavor, cocamidopropyl betaine, sodium gluconate, carbomer, sodium benzoate, Gantrez™ S-97, Poloxamer 407, xanthan gum, and sodium hydroxide. The test dentifrice had an RDA value of 88. The flavor was peppermint (provided by DSM-Firmenich), to match the most common flavor used in commercial dentifrices.

Plaque levels were assessed at baseline, then following a one minute single supervised brushing period with the assigned dentifrice, and then finally after 7 days of twice daily unsupervised use at home. For the post-brushing plaque evaluations, the clinical examiner was blinded to the product codes/identification and product assignments. Subjects were queried regarding adverse events both after the initial brushing episode and at the 7-day review.

### 2.2. Recruitment and Selection

A total of 82 subjects, aged between 19 and 69 years of age, were enrolled to ensure that approximately 80 subjects would complete the study. All subjects resided in Fort Wayne, IN, USA. The sample size was based on a power analysis. With 40 subjects per group, there is 80% power to detect a 0.634 effect size (mean difference between treatment groups divided by the pooled SD for the mean change from baseline) for a two-sided t-test with 5% type 1 error. This corresponds to detecting a large effect size, corresponding to a meaningful superiority in plaque reduction.

Informed consent was obtained from each subject prior to participation, in line with US Food and Drug Administration Good Clinical Practice guidelines for clinical studies. Information was given in both oral and written form, and subjects were given ample opportunity to inquire about details of the study prior to signing and dating the consent form. A copy of the signed consent form was given to each subject. This study was conducted according to the guidelines of the Declaration of Helsinki, and received ethics approval from the U.S. Investigational Review Board Inc, which is a US Department of Health and Human Services (DHHS) -registered independent ethics committee (#IRB00007024). The randomized controlled trial registration number is NCT06082869.

The inclusion criteria included having good general health (based on a medical history review), having a minimum of 18 natural teeth (excluding all those with gross caries, cervical abrasion and/or enamel abrasion, orthodontic bands, crowns, veneers, third molars, and implants). Subjects were required to have, a mean full-mouth pre-brushing plaque score of ≥2.00 at the baseline visit (based on the Soparkar modification of the Turesky modification of the Quigley and Hein plaque index) [22,23,24].

Subjects were required to not undergo dental prophylaxis or any other elective, non-emergency dental procedures (other than those provided for the study) at any time during the 7-day period of the investigation. Subjects also agreed to refrain from using any oral hygiene products (i.e., floss, mouthwash, etc.), other than the toothbrush and toothpaste supplied, for the 7-day period of the study. Subjects refrained from all oral hygiene procedures (including chewing gum) for 24 h, and from eating, drinking and smoking for 2 h prior to the two clinical visits of the study.

The exclusion criteria included: having undergone a periodontal treatment or having received a dental prophylaxis within 30 days prior to the baseline visit; pregnancy; breastfeeding; known adverse reactions, allergies or sensitivities to oral hygiene products or to any ingredient in the test materials; any physical limitations or restrictions that might preclude normal tooth brushing; the presence of fixed or removable orthodontic appliances, bridges, peri/oral piercings, or removable partial dentures; the use of antibiotics; the use of any medications with anticholinergic, sympathomimetic or vasoconstrictive actions on salivary glands (such as antidepressants, antipsychotics, anticholinergics, antihypertensives, antihistamines, and sedatives) [25,26,27] within 30 days prior to the study; the use of drugs that affect oral soft tissues within 30 days prior to the study, the presence of oral soft tissue pathology; the presence of severe periodontal disease, undergoing periodontal treatment; rampant dental caries; and the presence of extrinsic stain or calculus deposits that could interfere with plaque assessments.

### 2.3. Plaque Scoring

At the initial visit, each subject rinsed their mouth with an erythrosine disclosing solution. Plaque was scored on the facial and lingual tooth surfaces using the Soparkar modification of the Turesky modification of the Quigley and Hein plaque index. As mentioned earlier, all subjects were required to have a mean full-mouth pre-brushing plaque score of ≥2.00 at baseline to be eligible for the study. Plaque was scored in six areas per tooth (mesio-facial, mid-facial, disto-facial, mesio-lingual, mid-lingual, and disto-lingual) from 0 to 5, giving a maximum possible score of 30 per tooth. The scoring criteria were as follows: 0 = No plaque; 1 = Separate flecks or discontinuous band of plaque at the gingival (cervical) margin; 2 = Thin (up to 1 mm), continuous band of plaque at the gingival margin; 3 = Band of plaque wider than 1 mm but less than 1/3 of tooth surface area; 4 = Plaque covering 1/3 or more, but less than 2/3 of tooth surface area; and 5 = Plaque covering 2/3 or more of tooth surface area.

After randomization, subjects were provided with a toothbrush (Oral-B 35) [28] and then performed the first supervised brushing intervention using a known (weighed) amount of the designated dentifrice for one min, after which a second soft and hard tissue examination was completed. The purpose of this second examination was to identify any changes in color or texture, soft tissue abrasion, and any irregularities. After a second application of disclosing solution, a post-brushing assessment of plaque levels was undertaken. The plaque score at baseline was used to calculate the extent of plaque removal from the first brushing episode.

At the end of the initial visit, subjects were reminded to brush twice daily for the next week, with the toothbrush and dentifrice that they received. They were reminded to refrain from all oral hygiene (including chewing gum) for 24 h prior to their next visit and to refrain from eating, drinking, and smoking for 2 h. prior to their next visit. These instructions were repeated by telephone prior to the second visit. During the one-week period, subjects completed a toothbrushing diary. 

The second visit occurred 7 days later, and followed a similar workflow, with an initial soft and hard tissue examination, followed by disclosing and scoring plaque levels. These data would then later be compared to the baseline plaque levels at the start of the first visit. During the second visit, a second supervised 1 min brushing intervention occurred using the assigned dentifrice, after which there was a further soft and hard tissue examination, followed by disclosing and scoring plaque levels. All examinations in the study were performed by the same dental examiner, who was blinded to the treatment allocations. 

At both visits, the examining dentist assessed subjects for adverse changes, and also queried subjects regarding any adverse events.

### 2.4. Data Analysis

A plaque index score for each subject was calculated by adding all the individual plaque scores (six per tooth), and dividing this sum by the total number of measurements (number of teeth scored multiplied by six). Only the subjects who completed the 1-week test period and had no protocol violations were included in the efficacy analysis. 

The primary efficacy parameter was reduction in subject-wise mean plaque index scores from pre-brushing to post-brushing for all surfaces of the whole mouth. Within-treatment comparisons of the pre-brushing plaque scores to the post-brushing plaque scores were performed using paired t-tests. An unpaired t-test was used to compare treatment groups for the difference in the mean changes from baseline. 

Additionally, secondary efficacy parameters were assessed, namely the corresponding reduction in subject-wise mean plaque scores based on evaluations made on various subsets, including proximal, facial, lingual, gingival, lingual proximal, lingual gingival, posterior proximal, posterior facial proximal, posterior lingual proximal, posterior gingival, posterior facial gingival and posterior lingual gingival.

Continuous variables for demographic data were also evaluated using an unpaired (two-sample) t-test, while data for categorical variables were assessed using Fisher’s exact test. All statistical tests were two-sided and conducted at the 0.05 significance level. Statistical analyses were performed using SAS version 9.4 for Windows (SAS Institute, Sydney, Australia). Parametric statistical tests were used for continuous variables as data sets met the required assumptions including having normal distributions and comparable variances.

## 3. Results

### 3.1. Participants

After applying the selection criteria, a total of 82 subjects (63 females, 19 males) were enrolled, and all subjects completed the study, with 41 in each group. The mean age for all 82 subjects was 48.7 (range 19–69 years), and the test and control groups had similar mean ages and age ranges (50.9 years (range 24–69), and 46.5 years (range 19–67), respectively). There were no statistically significant differences between the two groups for any demographic variables (age, gender, race, and ethnicity), and likewise no significant differences in baseline whole mouth plaque scores (Crest 3.13 + 0.31 (SD), Protegera 3.02 + 0.32). No subjects had fixed or removable orthodontic appliances, bridges, peri/oral piercings, or removable partial dentures.

### 3.2. Plaque Removal

Data for plaque scores are shown in Table 1, while data for the primary outcome of whole mouth plaque reduction are shown in Table 2. Both dentifrices gave statistically significant reductions from the baseline levels (*p* < 0.001 for both); however, across all three assessments, the test dentifrice was three or more times more effective than the control (*p* < 0.001 for each). Thus, the null hypothesis was rejected.

Due to the accumulative effect of the test dentifrice lowering the pre-brushing plaque scores at day 7, plaque scores differed between the two treatments at the start of the day 7 visit. An analysis of covariance model was used for the between-treatment analysis to adjust for this difference, using the day 7 pre-brushing plaque score as the covariate, to improve the power and estimates of the treatment effects.

### 3.3. Site-Specific Effects

**Figure 3:**
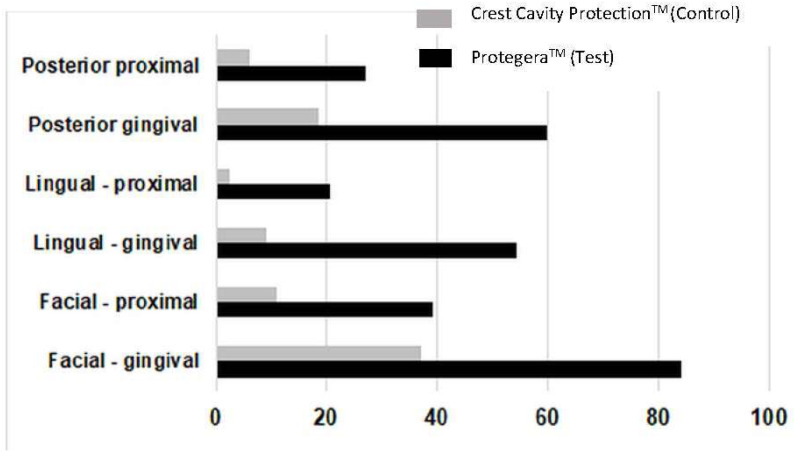
Plaque reduction (in percent) for one week of brushing. In all cases, the test (Protegera^TM^) dentifrice was superior to the control (Crest Cavity Protection^TM^) dentifrice (*p* < 0.001).

When secondary efficacy parameters were assessed, plaque removal for the test dentifrice was superior to the control at each of the three-time intervals (*p* < 0.001 for each) for all 14 subsets, including proximal, facial, lingual, and gingival (Table 2), as well as for lingual proximal, lingual gingival, posterior proximal, posterior facial proximal, posterior lingual proximal, posterior gingival, posterior facial gingival and posterior lingual gingival (Figure 3). Of note, the test dentifrice was superior for reducing dental plaque levels in the gingival aspect of teeth, with a 62.5% reduction on first use, a 56.3% reduction at day 7, and a 68.7% reduction from day 1 to day 7, all of which were superior to the positive control (*p* < 0.001). Data for the mean difference in plaque score between treatments (i.e., test dentifrice plaque score versus control dentifrice plaque score for the day 1 baseline to the day 7 post-brushing score) are summarized in Table 3.

### 3.4. Adverse Outcomes

No safety issues or adverse responses arose from the use of either dentifrice.

## 4. Discussion

The purpose of the present study was to assess the plaque removal efficacy and safety of a novel dentifrice containing micro-fibrillated cellulose and microfibrils of cellulose in individuals with moderate to high levels of dental plaque, immediately after 1 min of supervised use, following a week of twice daily use at home, and a second supervised brushing period at 7 days, and to compare this to a conventional dentifrice.

The results of this clinical study show that the test dentifrice based on MFC and tailored mechanical properties, was at least three times more effective in whole-mouth plaque reductions than the positive control dentifrice. As a result of superiority in all comparisons that were made, including reductions in whole mouth scores and all 14 site subsets, the null hypothesis of equivalence was rejected. Both dentifrices had the same levels of sodium fluoride and both contained surfactants. It is thus unlikely that antimicrobial actions of either of these components contributed to differences in performance. 

The results of the present study also show that the superiority of the cleaning action extends to sites of different types across the dentition, including areas involving the interproximal space, a region within the dentition that has limited accessibility to the bristles of a toothbrush. It is likely that the novel dentifrice can effectively transfer the forces of brushing to the dental plaque biofilm that is being removed without direct contact between the toothbrush bristles and the biofilm. This can occur since, under the forces of brushing, MFC fibers and fibrils are forced to flow into the interproximal space where they can then dislodge plaque deposits. Such a fluid-based cleaning action is an essential extension to concepts of dental plaque removal, given the well-established principle that physical plaque removal by a toothbrush, with or without a traditional dentifrice, is due to direct contact between the bristles and the dental plaque biofilm [8,9,13]. As mentioned earlier, with traditional tooth brushing methods, the dentifrice is rapidly diluted by stimulated saliva, and that limits the influence of bristles on dental plaque biofilms [14,15]. On the other hand, using a dentifrice with MFC can improve the plaque removal effect as the MFC network is less prone to breaking down from stimulated saliva [20], even when the same tooth brushing technique is being used. In the present study, the subjects did not change their brushing method (applied force or bristle angulation), but continued using their normal method. It is possible that changes in toothbrush design and in tooth brushing methods may enhance the effect seen from including MFC in a dentifrice, and these aspects deserve further study. Likewise, future studies could explore the effect of manual dexterity on cleaning performance.

An important consideration with a novel cleaning approach is that the method is safe for teeth and oral soft tissues [29,30,31]. The test toothpaste had an RDA of 88, and was designed to give a similar mouth feel and taste as regular dentifrices. It did not cause any abrasions of the oral soft or hard tissues, and no concerns or adverse effects were reported by the subjects. Given the short time scale of the present trial, longer clinical studies would be informative regarding safety aspects. Since the fiber complexes themselves have low abrasivity, issues such as abrasion of root surfaces are unlikely to arise. 

A further note is that the same toothbrush (Oral-B 35) was used with both products. It is feasible that different designs of toothbrushes could be explored to maximize the ability of the MFC to remove dental plaque deposits. Moreover, changes in the formulation of the dentifrice could be explored to provide some degree of chemical plaque control such as calculus control, or others. This may lead to even better performance when the dentifrice is used over extended periods of time.

The strengths of the study included that the two groups were well matched for dental plaque scores at the day 0 baseline visit, and for age, gender, race, and ethnicity. Additionally, the study was able to assess the effect of a single supervised brushing intervention (on day 1 and at day 7) and also the impact of 6 days of unsupervised brushing. A limitation of the study was that the brushing time across all aspects of the study was 1 min rather than the 2 min time which is used as a benchmark. A longer brushing time may alter the differences seen between the effectiveness of the test and control dentifrices. A further limitation is that the study time period for home use was relatively short. Longer-term studies should explore plaque removal over periods of 3 months or more.

## 5. Conclusions

The present clinical study shows that a dentifrice designed to maximize the cleaning action of micro-fibrillated cellulose (MFC) outperforms a traditional dentifrice when used with the same toothbrush. The overall gain in performance was at least threefold and up to fourfold when considering whole-mouth plaque scores and up to sevenfold when considering lingual plaque scores. No safety issues arose from the use of the novel dentifrice. These results indicate that including micro-fibrillated cellulose enhances plaque removal effectiveness, without causing adverse changes to oral soft tissues.

## 6. Patents

Authors M.E.L., J.M. and Y.T. are named inventors on a patent (U.S. patent 10,266,793), M.E.L. and A.P. are named inventors on patent application US 2021/0121386 A1, and M.E.L., A.P., J.M. and Y.T. are named inventors on patent application US 2021/0330557 A1 that are relevant to this clinical trial.

## Figures and Tables

**Table 1:** Whole mouth plaque scores at four time points.

	Whole Mouth—Test	Whole Mouth—Control
Baseline day 1	3.02 (0.32) (2.39–3.78)	3.13 (0.31) (2.52–3.83)
Post-brushing day 1	1.86 (0.38) (1.07–2.70)	2.75 (0.43) (2.81–3.72)
Day 7 pre-brushing	2.56 (0.35) (1.85–3.14)	3.06 (0.28) (2.43–3.54)
Post-brushing day 7	1.77 (0.34) (0.84–2.45)	2.76 (0.30) (2.13–3.31)

Data show means, standard deviations, and range for n = 41 per group.

**Table 2:** Site-specific reductions in dental plaque levels.

Parameter	Product	Day 1 *	Day 7 *	One Week Use *	Improvement
Whole mouth	Test	38.6%	30.9%	41.6%	
	Control	12.0%	9.6%	11.6%	4×
Gingival	Test	62.5%	56.3%	68.7%	
	Control	21.5%	20.3%	23.0%	3×
Proximal	Test	28.6%	22.4%	30.1%	
	Control	7.9%	5.0%	6.7%	5×
Facial	Test	49.5%	40.5%	52.2%	
	Control	19.5%	16.8%	18.6%	3×
Lingual	Test	27.7%	22.3%	31.0%	
	Control	4.4%	2.2%	4.5%	7×

* Data show mean reductions in plaque scores in percent for the three relevant time points (N = 41 subjects per group). For all analyses, the test dentifrice was superior to the control (*p* < 0.001). Day 1 = Day 1 baseline to post-brushing; Day 7 = Day 7 baseline to post-brushing; One week = Day 1 baseline to day 7 post-brushing, representing the impact of one week of unsupervised brushing at home. “Improvement” refers to plaque removal improvement of the test dentifrice as compared to the control one in terms of plaque removal percentages after one week of use.

**Table 3:** Plaque index difference between treatments at one week.

Subset	Difference ^1^
Whole Mouth	0.89 (0.77–1.01)
Gingival	1.19 (1.02–1.36)
Proximal	0.74 (0.62–0.87)
Facial	0.98 (0.80–1.17)
Lingual	0.80 (0.68–0.93)

^1^ Data show the mean difference in plaque score between the test and control groups for the day 1 baseline to the day 7 post-brushing score and the 95% confidence limits. All differences between treatments were statistically significant (*p* < 0.001).

## Data Availability

Data are available upon reasonable request to the corresponding author.
